# Are Marine Heatwaves Responsible for Mortalities of Farmed *Mytilus galloprovincialis*? A Pathophysiological Analysis of *Marteilia* Infected Mussels from Thermaikos Gulf, Greece

**DOI:** 10.3390/ani12202805

**Published:** 2022-10-17

**Authors:** Athanasios Lattos, Dimitrios K. Papadopoulos, Konstantinos Feidantsis, Dimitrios Karagiannis, Ioannis A. Giantsis, Basile Michaelidis

**Affiliations:** 1 Laboratory of Animal Physiology, Department of Zoology, Faculty of Science, School of Biology, Aristotle University of Thessaloniki, 541 24 Thessaloniki, Greece; 2 Laboratory of Ichthyology, Faculty of Veterinary Medicine, Aristotle University of Thessaloniki, P.O. Box 395, 541 24 Thessaloniki, Greece; 3 Department of Animal Science, Faculty of Agricultural Sciences, University of Western Macedonia, 531 00 Florina, Greece

**Keywords:** marteiliosis, bouchot mussel farming, histopathology, parasite molecular identification, defense response

## Abstract

**Simple Summary:**

Climate change effects strongly and negatively influence the health and productivity of marine bivalves and particularly bivalve aquaculture. It is therefore of critical importance to study the physiological response of bivalves against marine heatwaves under field conditions. Thus, in the present study, we evaluated the pathophysiological pathways of the Mediterranean mussel originating from mortality events attributed to extreme seawater temperatures in the context of *Marteilia refrigens* infection. Our study is focused on the Thermaikos Gulf, the most important mussel cultivation area in Greece, comparing the traditional bouchot-like mussel farming system with the modern long-line system. Heatwaves increased all examined molecular, biochemical and pathophysiological markers in *M. galloprovincialis* mussels, which were reinforced in Marteilia infected individuals. Therefore, these results enlighten us on the biological impacts of heatwaves, providing valuable information regarding the underlying mechanisms. Thus, insights for the future management of the marine aquatic sector could be provided and subsequently address measures for the decrease or elimination of this phenomenon and restoration of marine production.

**Abstract:**

Marine heatwaves (excessive seawater temperature increases) pose high risk to bivalves’ health and farming. The seawater temperature increase is responsible for various pathogen population expansions causing intense stress to marine organisms. Since the majority of knowledge so far derives from laboratory experiments, it is crucial to investigate stress responses in field conditions in order to understand the mechanisms leading to bivalves’ mortality events after exposure to temperature extremes. Thus, we evaluated the pathophysiological response of the Mediterranean mussel *Mytilus galloprovincialis* originating from mortality events enhanced by intense heatwaves in Thermaikos Gulf, north Greece, along with *Marteilia refrigens* infection. Mussels that have been exposed to high environmental stressors such as high temperature were examined for various molecular and biochemical markers, such as *hsp70*, *bax*, *bcl-2*, *irak4* and *traf6* gene expression, as well as the enzymatic activity of the hsp70, hsp90, bax, bcl-2, cleaved caspases, TNFa and ll-6 proteins. Furthermore, histopathology and molecular positivity to *Marteilia* sp. were addressed and correlated with the gene expression results. Our findings elucidate the molecular and biochemical pathways leading to mortality in farmed mussels in the context of *Marteilia* infection, which according to the results is multiplied by heatwaves causing a significant increase in pathophysiological markers.

## 1. Introduction

Aquaculture continues to thrive among food industry production sectors, expanding faster than any other livestock sector worldwide during recent decades [1,2,3]. Particularly, bivalve farming represents an important marine aquaculture activity worldwide, raising production from nearly 1 million tons in 1950 to 14.54 million tons in 2013, with an annual value of more than USD 15 billion [4,5]. In Greece, although oyster and clam farming have been recently licensed, bivalve cultivation is still represented exclusively by the Mediterranean mussel *Mytilus galloprovincialis* (Lamarck, 1819) farming [6,7,8]. Mussel culture is depending on wild stocks since in the process of mussel farming, spat (juveniles) is collected from collector ropes or traps and transferred to neighboring mussel farming areas for cultivation, or occasionally to longer distances [9]. Thus, compared with fish or shrimp farming, bivalve culture depends on adequate eutrophication of the culture site and has to have less impact on the local ecosystems [10,11]. In contrast to fish and shrimp aquaculture, capture-based mussel culture is totally exposed to environmental factors in the whole process of farming [12].

Despite the positive impacts of mussel farming on the national economy and local ecosystems [6], human pressures including anthropogenic climate change tend to cause complications that result in a reduction of productivity. Rapid fluctuations of abiotic factors, as an impact of climate change, negatively affect production, leading to financial distress in culture farms [13]. Acute temperature increases in surface sea water are one of the driving impacts of climate change in aquatic ecosystems and thus have been studied intensively in recent decades, having been found to correlate with the down-regulation of both physiological and immunological functions in marine bivalves [14,15]. Specifically, thermal stress has been correlated with increased oxidative stress in marine invertebrates [16,17,18,19], the down-regulation of metabolic markers and anaerobiosis in marine bivalves [20]. Concerning immune responses, increased temperatures result in high levels of phagocytic ability and total number of hemocytes in marine bivalves [15,21,22,23]. Furthermore, in the context of climate change, increasing temperature has been shown to induce several cellular signaling pathways, including that of apoptosis [24,25,26].

The temperature rise also does not only affect marine species’ physiological and immunological processes but also affects the spread of microorganisms that are endemic to marine ecosystems [27]. Despite the beneficial roles of the microorganisms [27], many of them, which have been described as temperature dependent, constitute opportunistic pathogens for marine bivalves [28]. Several recent studies have confirmed the role of temperature in emerging infectious diseases resulting from vibrios [29]. Specifically, *Vibrio tubiashii* was initially described as a temperature-dependent, hard-shell clam pathogen [28,30]. *Vibrio tapetis* was isolated for the first time in 1987 in France and later on other Mediterranean coasts and in Norway, Korea and Japan in mortality events in the *Ruditapes philippinarum* clam [31,32,33]. Temperature and salinity have been considered to be inhibitors in the growth of this bivalve pathogen at temperatures above 22 °C and salinities greater than 5% [34]. *Vibrio mediterranei* were isolated and correlated with dead *Pinna nobilis* individuals in an experiment conducted in order to describe disease outbreak in stabled individuals [35,36]. Although the latter pathogen triggered its virulence mechanisms at temperatures above 24 °C, it was also present in *P. nobilis* natural populations at lower temperatures without triggering mortalities, similar to those exhibited at 24 °C [35,36,37].

Despite the bacterial infectious diseases in bivalve populations, protozoan parasites can be also influenced by climate change-induced temperature increase [38,39,40]. Among the *Perkinsus* spp., *Perkinsus marinus* and *Perkinsus olseni* have been associated with mortality events in *Crassostrea virginica* and *Ruditapes decussatus* and *Ruditapes philippinarum* clams [40,41]. These parasites seem to be favored by higher temperatures and secondarily by other external stressors such as food availability [40,42]. Regarding Haplosporidian parasites (the phylum comprises over 40 described species), mostly *Bonamia* spp. and *Haplosporidium* spp.have been implicated in mortalities in marine bivalve populations [43]. Both genera have been strongly correlated to temperature and salinity, which affect their distribution patterns and seasonal prevalence [38,44]. *Martelia* spp., which was identified for the first time in *Ostrea edulis* oysters in France, is the main protozoan species identified in Greek mussel culture sites in the past [45,46,47]. Similar to the above, increased temperature through the year seems to be a driving factor implicated in the life cycle of this parasite [48,49].

**Figure 1:**
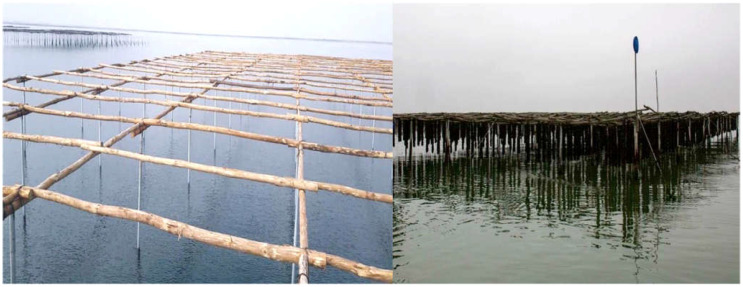
The hanging park bouchot type traditional technique applied in mussel farming in Axios River Delta (Kymina, Thermaikos Gulf, north Greece).

Concerning bivalve farming in Greece, Thermaikos Gulf is the culture site with the highest productivity of mussels, representing 90% of the total mussel production, while 80% is exported to other European countries [50]. However, in 2020 and 2021, reinforced by the COVID-19 pandemic situation that delayed harvesting, mass mortality events in Greek mussels farms resulted in the collapse of productivity, reaching 100% loss of the production in northern Greece’s farms. Mortalities began with the rise of the temperature and eventually killed the total of the population when surface sea water temperature reached 30 °C in the hanging park “bouchot type” culture, a traditional culture technique, similar to the French bouchot culture as well as to raft culture (Figure 1), with the modification that the vertical poles are embedded in the shallow waters (up to 1.5–2 m) of the Axios River Delta (north Greece). This technique provides the advantage to the farmers of controlling the mussel sleeves more easily and using smaller boats than in the modern longline technique that utilizes floats and needs larger boats, but at the same time mussels are more vulnerable to abiotic factors due to the shallow waters.

Keeping in mind the lack of complete knowledge concerning the physiological response of infected or uninfected *M. galloprovincialis* against various pathogens during intense heat waves, the objective of this study is the investigation of this species’ pathophysiological responses originating from mortality events, in order to identify the causative agent(s) of the mortalities. To assess the above, *hsp70* gene expression as well as Hsp70 and Hsp90 induction levels, apoptotic indicators such as Bax/Bcl-2 ratio (transcription and translation levels) and cleaved caspase levels, immunological indicators such as the mRNA expression of *irak4* and *traf6* genes, and TNFα (tumor necrosis factor) and Il-6 (interleukin 6) levels, as well as histopathology and molecular positivity to *Marteilia* sp., were addressed. In an effort also to correlate the expression of stress- and immunology-related genes at both mRNA and protein levels, the expression levels were compared between infected and non-infected individuals regardless of the parasite load. It should also be noted that although co-infections in aquatic species are frequent [37], mussels are mainly affected by *Marteilia refrigens*, which constitutes the etiological agent of several mortality events in the past [47], and thus, only this pathogen was examined.

## 2. Materials and Methods

### 2.1. Sampling Procedure

**Figure 2:**
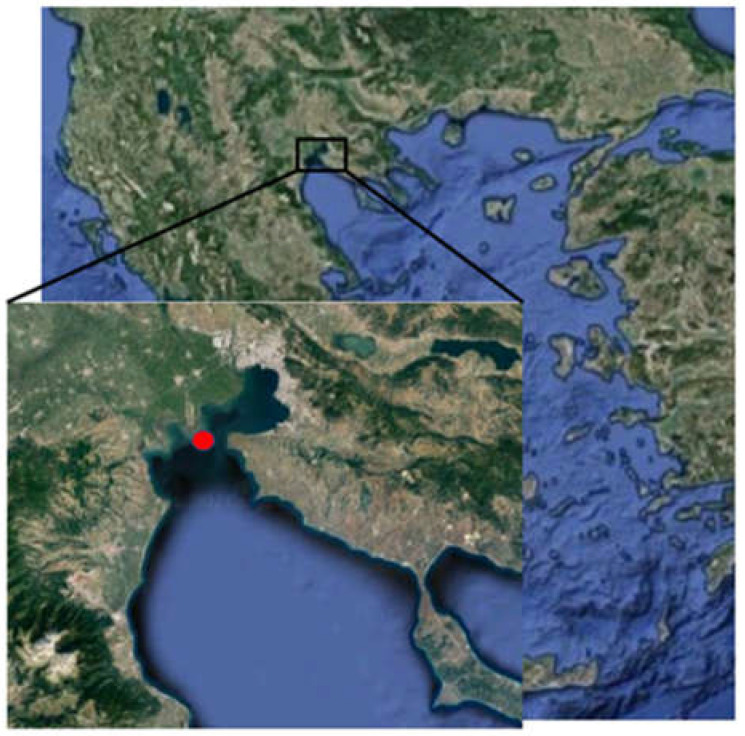
Balcanic peninsula including Greece. Sampling site (red dot) Kimina site (40.474678, 22.723543) of both sampling techniques.

Sampling of adult *M. galloprovincialis* [22.43 ± 3.21 g (mean ± SD), shell length 5.62 ± 0.35 cm and shell width 2.89 ± 0.11 cm] was conducted in both of the culture processes (traditional culture-Bouchot (TC) and long-line system (LC)) three times during the period of mortalities in the area of Thermaikos Gulf (Figure 2). More specifically, in 2022, at the beginning of July, when surface sea water temperature reached 28 °C, mortalities were also observed. Large-scale precipitation during the next 3 days (8 July 2022–11 July 2022) decreased surface sea water temperature to 26 °C. Afterwards, on 25 July 2022, temperature increased to 28.2 °C, resulting again in mortalities in both culture processes. These mortalities continued their increase until they reached 100% in bouchot culture on 1 August 2022, when surface sea water temperature reached 29.5 °C.

Ten individuals from each culture process were sampled in each effort in order to evaluate the physiological condition of the cultured mussels. Half of the digestive gland and mantle were extracted from each sample, stored in 1.5 mL eppendorfs and kept in liquid nitrogen immediately after the dissection process. Thereafter, samples were stored at −80 °C until further biochemical analyses.

### 2.2. Histopathological Procedure

The other half of the digestive gland was fixed immediately in Davidson fixative agent for the histological scanning for potential pathogen identification, according to Shaw and Battle [51]. After dehydration through graded alcohols, *M. galloprovincialis* tissues were embedded in paraffin wax and sectioned in 5 μm using a rotary microtome. Afterwards, staining of the samples was conducted using hematoxylin and eosin according to the protocol of Howard et al. [52].

### 2.3. SDS-PAGE/Immunoblot and Dot Blot Analysis

#### 2.3.1. Preparation of Tissue Samples

Tissue samples were homogenized (1/3 *w*/*v*) in cold lysis buffer (20mM β-glycerophosphate, 50 mM NaF, 2 mM EDTA, 20 mM Hepes, 0.2 mM Na_3_VO_4_, 10 mM benzamidine, pH 7, 200 μM leupeptin, 10 μΜ trans-epoxy succinyl-L-leucylamido-(4-guanidino)butane, 5 mM dithiotheitol, 300 μΜ phenyl methyl sulfonyl fluoride (PMSF), 50 μg mL^−1^ pepstatin and 1% *v*/*v* Triton X-100). After a 30-min extraction on ice, samples were centrifuged (10,000× *g*, 10 min, 4 °C), and the supernatants were boiled (3/1 *v*/*v*) with sample buffer (330 mM Tris-HCl, 13% *v*/*v* glycerol, 133 mM DTT, 10% *w*/*v* SDS, 0.2% *w*/*v* bromophenol blue). Protein concentrations were determined using the BioRad protein assay.

#### 2.3.2. SDS-PAGE/Immunoblot

Indicators of the heat shock response (HSR) and immunological and apoptotic pathways were determined in mantle samples according to well-established protocols for SDS-PAGE/immunoblot analysis. Specifically, equivalent amounts of proteins (50 μg) were separated on 10% (*w*/*v*) acrylamide and 0.275% (*w*/*v*) bisacrylamide slab gels and transferred electrophoretically onto nitrocellulose membranes (0.45 μm, Schleicher & Schuell, Keene, NH 03431, USA). Nonspecific binding sites on the membranes were blocked with 5% (*w*/*v*) nonfat milk in TBST (20 mM Tris-HCl, pH 7.5, 137 mM NaCl, 0.1% (*v*/*v*) Tween 20) for 30 min at room temperature. Nitrocellulose membranes resulting from the above procedure were subjected to overnight incubation with: monoclonal mouse anti-hsp70 (H5147, Sigma, Darmstadt, Germany), monoclonal mouse anti-hsp90 (H1775, Sigma, Darmstadt, Germany), anti-IL-6 (CSB-PA06757A0Rb, Cusabio, Houston, TX, USA), anti-TNFα (CSB-PA07427A0Rb, Cusabio, Houston, TX, USA), anti-Bcl2 (7973, Abcam, Cambridge, UK) and anti-Bax (B-9) (2772, Cell Signaling, Beverly, MA, USA). Quality transfer and protein loading were assured by Ponceau stain and actin (anti-β actin 3700, Cell Signaling, Beverly, MA, USA) (data not shown). Antibodies were diluted as recommended by the manufacturer’s guidelines. After washing in TBST (3 periods, 5 min each time), the blots were incubated with horseradish peroxidase-linked secondary antibodies and washed again in TBST (3 periods, 5 min each time), and the bands were detected using enhanced chemiluminescence (Chemicon, Darmstadt, Germany) with exposure to Fuji Medical X-ray films. Films were quantified by laser-scanning densitometry (GelPro Analyzer Software, GraphPad, San Diego, CA, USA).

#### 2.3.3. Dot Blot Analysis

Cleaved caspase levels were determined in mantle and PAM samples with the employment of a dot blot apparatus. Specifically, samples were diluted to a concentration of 5 μg mL^−1^ in a saline solution (150 mM NaCl); 100 μL volumes were loaded onto a pre-soaked nitrocellulose membrane (0.45 μm) in a dot blot vacuum (BioRad, Hercules, CA, USA) and gravity fed through the membrane. The membrane was blocked with 5% (*w*/*v*) nonfat milk in TBST [20 mM Tris-HCl, pH 7.5, 137 mM NaCl, 0.1% (*v*/*v*) Tween 20] for 30 min at room temperature. The resulting nitrocellulose membrane was subjected to overnight incubation with anti-cleaved caspase antibody (Cat. No.8698 Cell Signaling, Beverly, MA, USA). Antibodies were diluted as recommended by the manufacturer’s guidelines. After washing in TBST (3 periods, 5 min each time), the dots were incubated with horseradish peroxidase-linked secondary antibodies and washed again in TBST (3 periods, 5 min each time), and the dots were detected using enhanced chemiluminescence (Chemicon, Darmstadt, Germany) with exposure to Fuji Medical X-ray films. Films were quantified by laser-scanning densitometry (GelPro Analyzer Software, GraphPad, San Diego, CA, USA).

### 2.4. Molecular Identification of the Pathogen

For the molecular identification of the *Marteilia* protozoan pathogen, a small piece of approximately 25 mg of the homogenized digestive gland and mantle tissue was subjected to DNA extraction, using the Nucleospin Tissue DNA extraction kit (Macherey Nagel, Düren, Germany), following the manufacturer’s guidelines. The primer pair SS2/SAS2 was used in a 10 μL volume PCR for each individual, containing 1 μL extracted DNA of approximately 50 ng/μL concentration, 0.3 pmol of each primer, 5 μL FastGene^®^ Taq ReadyMix (2×) and water up to the final volume of 10 μL. Conditions were 95 °C for 3 min and then 94 °C for 30 s, 52 °C for 40 s and 72 °C for 45 s, repeated 36 times and followed by an extension step of 5 min at 72 °C. PCR products were visualized in an agarose gel after electrophoresis stained with ethidium bromide. A previously identified *Marteilis refrigens* sample [53] was utilized in each reaction as a positive control. Positive samples were cleaned using the NucleoSpin Gel and PCR Clean-up kit (Macherey Nagel, Düren, Germany) and bidirectionally sequenced for identification of the species.

### 2.5. RNA Extraction and cDNA Synthesis

Total RNA was extracted using NucleoZOL reagent (Macherey-Nagel, Düren, Germany) according to the manufacturer’s instructions. The optional phase separation step was not carried out, but all the other steps followed the protocol. Briefly, 50 mg of mantle tissue of each mussel was manually homogenized by pestling in 500 μL NucleoZOL, and RNAase-free water was added to the lysate. After shaking, samples were centrifuged, and isopropanol was added to the supernatant for RNA precipitation. Subsequently, samples were centrifuged, and two ethanol washes were performed. The washed RNA pellet was diluted in 60 μL nuclease-free water. Total RNA was kept at −80 °C. RNA quality and concentration were determined on a Quawell UV-Vis 5000 spectrophotometer (Quawell Technology, San Jose, CA, USA). Approximately 500 ng of total RNA of each sample were reverse transcribed, using a PrimeScript kit (Takara, Kusatsu, Shiga, Japan) and the oligodT primers, according to the manufacturer’s protocol. cDNA concentration was measured and the samples were kept at −20 °C, until the qPCR application.

### 2.6. Analysis of Gene Expression with Quantitative PCR

Five genes, heat shock protein 70 (hsp70), bcl-2 associated protein X (bax), B-cell lymphoma 2 (bcl-2), Interleukin-1 receptor-associated kinase-4 (irak4) and TNF receptor-associated factor 6 (traf6) were selected for gene expression analysis. The gene-specific primers used in this study are listed in Table 1. The comparative CT method (2^−ΔΔCT^) was applied to quantify the relative expression level of the genes, using the mantle cDNA of animals collected on the 26th of May as the control samples. Expression of target genes was normalized with the selected reference gene (endogenous control), elongation factor 1 alpha, which was more stable than the beta actin gene. For every gene, three different specimens from each group of mussels were run in real-time PCR. PCR reactions were conducted using KAPA SYBR^®^ FAST qPCR Master Mix (2× kit, in 10 μL total volume. Each reaction contained 10 ng of mussel cDNA as template, 5 μL of KAPA SYBR^®^ FAST qPCR Master Mix (2×), 2 μΜ of each primer and PCR-grade water up to 10 μL. Runs were performed in an Eco 48 Real-time PCR thermocycler (Illumina, San Diego, CA, USA) in 48-cell microplates.

### 2.7. Statistical Analysis

One-way analysis of variance (ANOVA) (GraphPad Instat 3.0) followed by Bonferroni post hoc analysis was employed to test for significance at *p* < 0.05 (5%) between all experimental groups examined herein. Since normality tests have little power to test the homogeneity of data for small sample sizes (such as the ones described herein: *n* = 5), Friedman’s nonparametric test and Dunn’s post hoc test were applied. Two-way ANOVA (GraphPad Prism 5.0) with different aquaculture techniques and *Martelia* spp. presence as fixed factors was employed to test for the significance of factor interactions.

## 3. Results

### 3.1. Histopathology and Molecular Identification of the Pathogen

**Figure 3:**
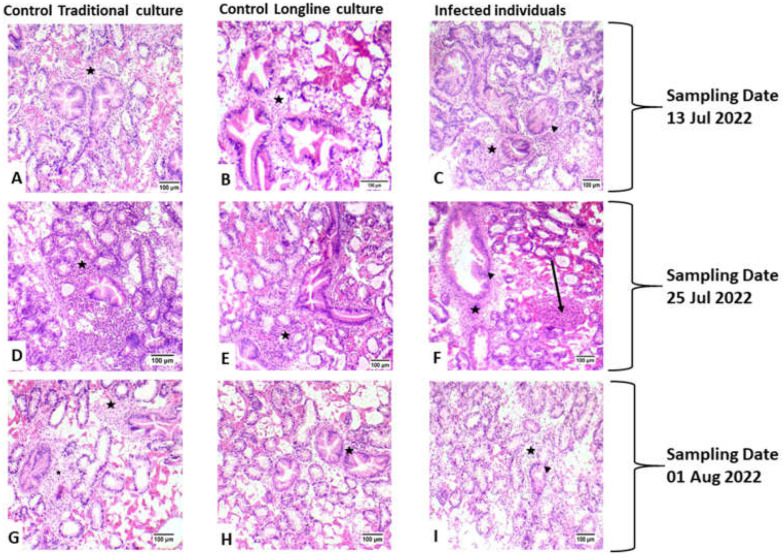
Histological display of sampled *M. galloprovincialis* individuals. (**A**–**C**) represent samples from the first sampling (13 July 2022), (**D**–**F**) represent samples from the second sampling (25 July 2022) and (**G**–**I**) are from the third sampling (1 August 2022). Regardless of the culture type or the health condition of the samples, all samples presented heavy hemocyte infiltration (star), while in samples infected with *Marteilia refringens* nodular type exhibited inflammation (arrow) and epithelial tissue degeneration (arrowhead) [H&E staining (×20 Objective)].

**Figure 4:**
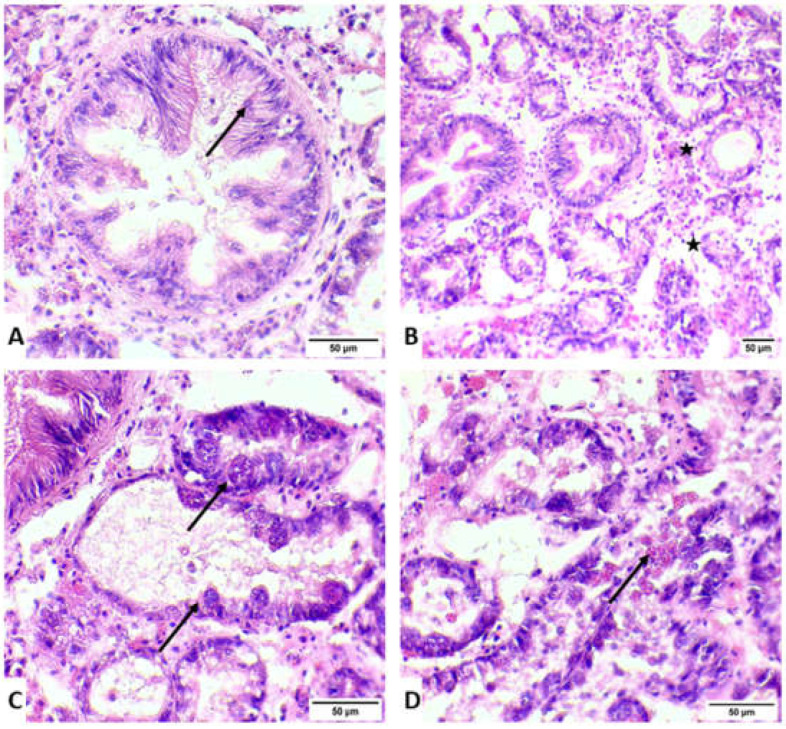
Histological display of *Marteilia refringens* life stages in digestive glands of *M. galloprovincialis*. (**A**) Histological display of a nurse cell in the digestive tubule epithelium (arrow). (**B**) Strong hemocyte infiltration of the digestive gland (star). (**C**) Sporangia life stage of *M. refringens* inside the digestive gland epithelium (arrows). (**D**) Sporangiospore development in the digestive gland (arrow). H&E ×40, ×20, ×40, ×40.

The histopathological examination documented the presence of developmental stages and mature stages in epithelium of digestive glands of *M. galloprovincialis*. Atrophic development was documented in the epithelium of digestive glands in samples infected with *Marteilia refringens* (Figure 3 and Figure 4). Initial stages (nurse cells-arrow) of *M. refringens* sporulation were detected in the epithelium of digestive tubules (Figure 3A), while mature stages were detected in the epithelial cells of digestive glands (Figure 3B). Atrophic epithelium along with damage in the epithelium indicates the release of sporangia from the lumen of digestive tubules (Figure 3C,D). Strong hemocytic (star) infiltration was observed in all samples regardless of *M. refringens* infection, although inflammation was observed in samples infected with *M. refringens*. The presence of the pathogen in the histopathologically positive samples was confirmed by molecular examination, which was identified as *Marteilia refringens* after BLAST searches of the derived sequences, exhibiting 100% similarity with other 18S SSU rRNA *M. refrigens* haplotypes hosting *M. galloprovincialis* and *Ostrea edulis* available in the GenBank database.

**Figure 5:**
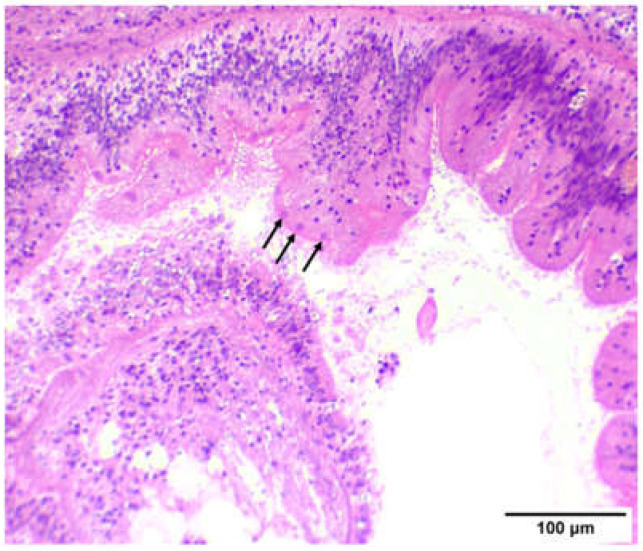
Young stages of *M. refringens* (arrows) within the epithelium of *M. galloprovincialis* digestive tract. H&E staining (×20 Objective).

In the sampling of 13 July 2022, no infected individuals were detected in LC, indicating a 0% prevalence rate, while in TC, 1 sample was detected with marteliosis and was characterized as heavily infected. In the sampling of 25 July 2022, the prevalence rate of marteliosis was 20% in both culture types; in both culture types, infected individuals were divided into light versus heavy infection rate. Finally, in the sampling of 1 August 2022, *M. refringens* was detected only in LC (Table 2). The severity of *M. refringens* infection was classified according to the histopathological results. Samples with a light infection rate were characterized by the presence of young stages of the protozoan parasite in the epithelium of the stomach and digestive tract (Figure 5), and samples with moderate infection rate were characterized by mature parasite cells in the digestive tubules, while samples with heavy infection rate were characterized by excessive presence of the parasite in all stages and heavy degeneration processes in the digestive tubules. Both lightly and heavily infected by *M. refringens* individuals showed similarly increased biochemical responses presented later, compared with the non-infected individuals. Likewise, no obvious correlation could be established between the rate of infection and the culture type (LC and TC)

### 3.2. Heat Shock Response

**Figure 6:**
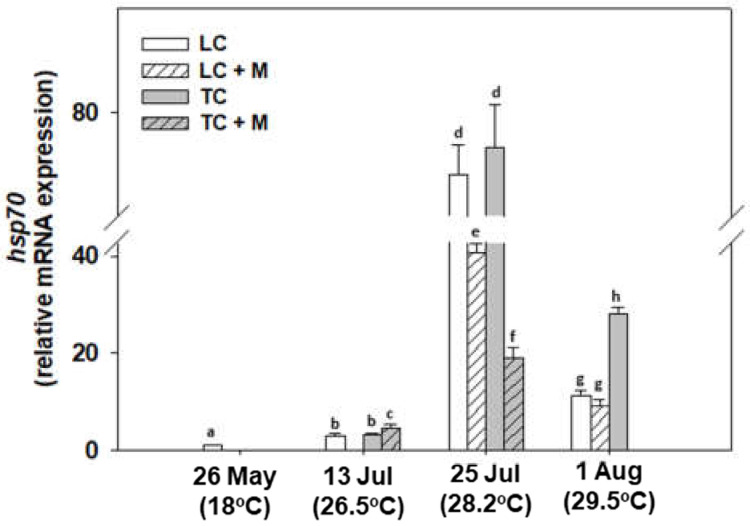
Relative *hsp70* mRNA expression levels in the mantle of *M. galloprovincialis* specimens, either infected with *Martelia* spp. (M) or not. Samples were collected from Thermaikos Gulf during a summer heatwave both from traditional culture-Bouchot (TC) and long-line system (LC). Values represent means ± SD; *n* = 5 preparations from different animals. Lower case letters indicate statistically significant differences (*p* < 0.05) between samples.

A generally increasing pattern was observed in *hsp70* gene expression, the levels of which increased in all samplings compared with the one in May. Specifically, the 25 July exhibited the highest values. While only on 1 August was TC statistically higher than LC, in general, samples infected by *Martelia* spp.exhibited lower *hsp70* expression levels compared with non-infected individuals (Figure 6).

**Figure 7:**
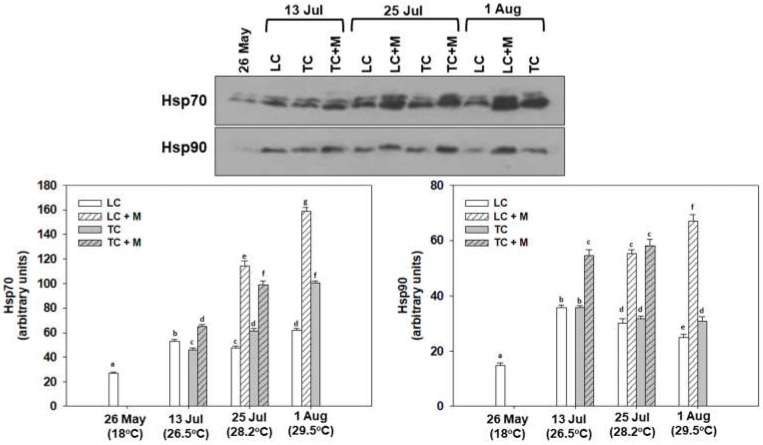
Hsp70 and Hsp90 levels in the mantle of *M. galloprovincialis* specimens, either infected by *Martelia* spp. (M) or not. Samples were collected from Thermaikos gulf during a summer heatwave both from traditional culture-Bouchot (TC) and long-line system (LC). Representative blots are shown. Values represent means ± SD; *n* = 5 preparations from different animals. Lower case letters indicate statistically significant differences (*p* < 0.05) between samples. Original blots: Figure S1.

Hsp70 and Hsp90 induction levels followed a similar pattern to that of the *hsp70* gene expression regarding levels compared with the sampling from May. Specifically, samplings on 13 and 25 July and 1 August exhibited statistically significant increases in both Hsp70 and Hsp90 compared with May. Non-infected individuals exhibited higher Hsp70 levels in August and higher Hsp90 levels on 13 July. Regarding the different culture techniques, while Hsp90 exhibited no significant differences, interestingly, TC Hsp 70 levels were increased compared with LC in the 25 July and 1 August samplings. Both proteins however, exhibited statistically significant increased levels in *Martelia* spp.-infected individuals compared with non-infected at all sampling points (Figure 7).

### 3.3. Apoptosis

**Figure 8:**
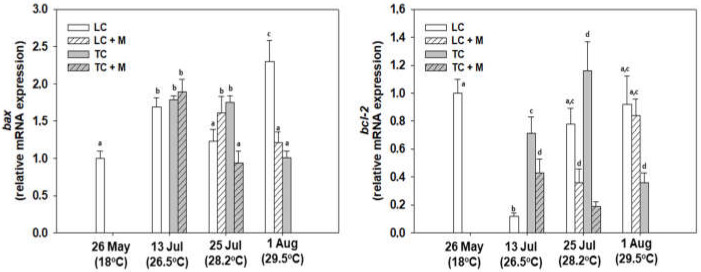
Relative bax and bcl-2 mRNA expression levels in the mantle of *M. galloprovincialis* specimens, either infected by *Martelia* spp. (M) or not. Samples were collected from Thermaikos gulf during a summer heatwave both from traditional culture-Bouchot (TC) and long-line system (LC). Values represent means ± SD; *n* = 5 preparations from different animals. Lower case letters indicate statistically significant differences (*p* < 0.05) between samples.

Regarding *bax* gene expression, its levels increased in all samplings compared with May, exhibiting the effect of the thermal stress. However, significant differences between TC and LC were only observed on 25 July and 1 August (TC > LC and LC > TC, respectively). Interestingly, individuals infected with *Martelia* spp. exhibited the same or lower levels of *bax* gene expression compared with non-infected individuals (Figure 8). 

On the other hand, *bcl-2* gene expression exhibited the same or lower levels compared with the sampling from May. The latter exhibits the effect of thermal stress on the suppression of the anti-apoptotic *bcl-2* gene expression. While in both July samplings, TC was significantly higher compared to LC, interestingly, an opposite pattern was observed on 1 August. Similar to *bax* gene expression, *Martelia*-positive individuals exhibited the same or lower levels of *bcl-2* gene expression compared with non-infected individuals (Figure 8).

**Figure 9:**
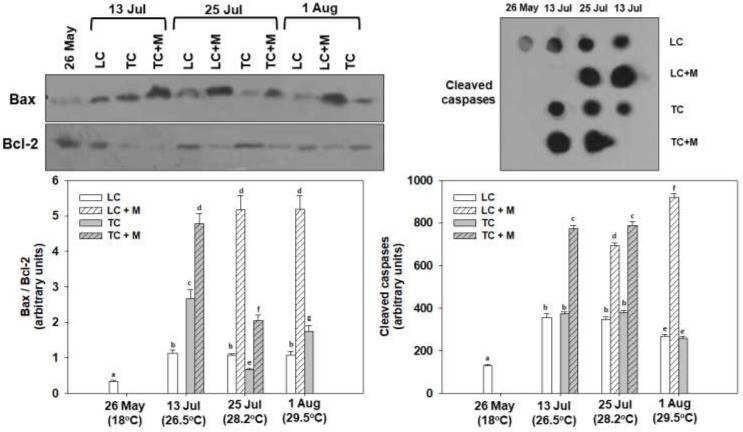
Bax/Bcl-2 ratios and cleaved caspase levels in the mantle of *M. galloprovincialis* specimens, either infected by *Martelia* spp. (M) or not. Samples were collected from Thermaikos Gulf during a summer heatwave both from traditional culture-Bouchot (TC) and long-line system (LC). Representative blots and dots are shown. Values represent means ± SD; *n* = 5 preparations from different animals. Lower case letters indicate statistically significant differences (*p* < 0.05) between samples. Original blots: Figures S2 and S3.

Bax/Bcl-2 ratio exhibited statistically significant increased levels at all sampling points compared with May, indicating increased apoptotic processes. TC was higher on 13 July and 1 August compared with LC, while the opposite pattern was exhibited on the 25 July. All *Martelia* spp.-infected individuals exhibited increased Bax/Bcl-2 ratio levels compared with non-infected individuals. While LC non-infected individuals exhibited no differences from 13 July to August, TC non-infected individuals exhibited their highest levels on 13 July (Figure 9).

Cleaved caspase levels, determining the apoptotic fate of the cell, were also statistically higher at all sampling points compared with May, depicting the heatwave’s devastating effect. While no statistical differences were observed between LC and TC non-infected individuals, 13 and 25 July exhibited higher caspases levels compared with 1 August. Again, all *Martelia* spp.-infected individuals exhibited increased cleaved caspase conjugate levels compared with non-infected individuals (Figure 9).

### 3.4. Immunology

**Figure 10:**
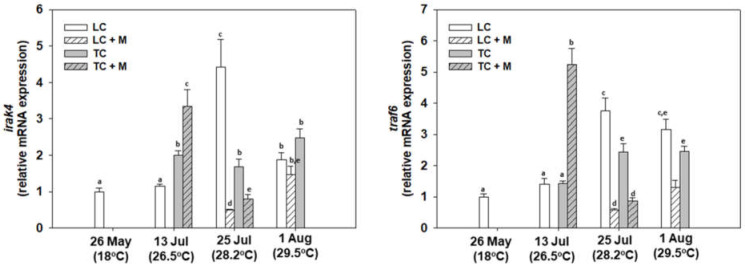
Relative *irak4* and *traf6* mRNA expression levels in the mantle of *M. galloprovincialis* specimens, either infected by *Martelia* spp. (M) or not. Samples were collected from Thermaikos Gulf during a summer heatwave both from traditional culture-Bouchot (TC) and long-line system (LC). Values represent means ± SD; *n* = 5 preparations from different animals. Lower case letters indicate statistically significant differences (*p* < 0.05) between samples.

In general, *irak4* gene expression levels increased in all samplings compared with May. Once again, the increase in these genes related to immunological processes during the heatwave underline that this phenomenon placed significant stress on the mussels. Regarding non-infected individuals, the heatwave differentially affected LC and TC. Individuals infected with *Martelia* spp. exhibited increased levels only on 13 July compared with non-infected individuals, while those on 25 July and 1 August exhibited the opposite pattern, which was also observed regarding the *traf6* gene expression levels (Figure 8). Likewise, *traf6* gene expression exhibited the same seasonal pattern of expression as that of *irak4* (Figure 10).

**Figure 11:**
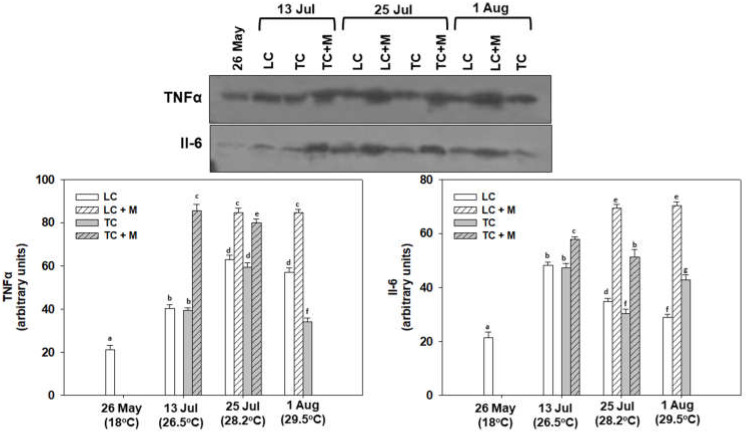
TNFα and Il-6 levels in the mantle of *M. galloprovincialis* specimens, either infected by *Martelia* spp. (M) or not. Samples were collected from Thermaikos Gulf during a summer heatwave both from traditional culture-Bouchot (TC) and long-line system (LC). Representative blots are shown. Values represent means ± SD; *n* = 5 preparations from different animals. Lower case letters indicate statistically significant differences (*p* < 0.05) between samples. Original blots: Figure S4.

As expected, post-translational immunological indicators were also statistically increased during the heatwave. Specifically, TNFα levels exhibited statistically significantly increased levels at all sampling points compared with May, with the highest levels observed on 25 July. While no statistical differences were observed between LC and TC non-infected individuals on 13 and 25 July, on 1 August, LC was statistically higher than TC. All *Martelia* spp.-infected individuals exhibited increased TNFα and Il-6 levels compared with non-infected individuals (Figure 9). Il-6 levels exhibited statistically significant increased levels at all sampling points compared to May, with the highest levels observed on 13 July. While no statistical differences were observed between LC and TC non-infected individuals on 13 and 25 July, on 1 August TC was statistically higher than LC (Figure 11).

## 4. Discussion

To the best of our knowledge, this study represents the first attempt directly performed in the field to elucidate the mortality of cultured mussels *M. galloprovincialis* due to synergistic effects of marteliosis and heatwave exposure in Thermaikos Gulf. Marteliosis or ‘’Aber disease’’ has been correlated with mortalities in Greek shellfish populations in the past in *M. galloprovincialis* and *O. edulis* [46,58]. However, disease etiology is considered complex in the concomitant presence of microorganisms and the ongoing rapid climate changes [59,60]. *M. refringens* dynamics in Mediterranean marine bivalve species are correlated with temperature rise [61]. Although the multiplication and transmission of the parasite are triggered at temperatures above 17 °C, many studies have indicated that the parasite seems to be abundant at mature stages also when exposed to winter temperatures [61,62,63]. The presence of protozoan parasites and specifically *M. refringens* has been associated many times with down-regulation in the physiology and immune responses of marine bivalves [60,64,65]. Apart from the presence of pathogenic microorganisms that affect the physiology of the marine bivalves, abiotic factors also possess a key role in the physiology of the marine organisms [4]. Acute and prolonged thermal stress resulting from marine heatwaves have been related to higher energy costs due to increased oxygen consumption [66] and higher antioxidant responses due to increased ROS production (at temperatures beyond 26 °C) [67,68]. High, steady ROS production in combination with higher energy costs may trigger mortality in *M. galloprovincialis* [19]. Regarding immune responses of marine bivalves, temperature fundamentally affects their immune system. For example, low total hemocyte count was demonstrated when *Chamelea gallina* was exposed to increased temperatures [69]. Moreover, phagocytic activity was also induced by higher temperatures alongside with lysozyme activity at increased temperatures [69].

Concerning the results of the current study, histopathological analysis documented induced hemocytic infiltration at all exposure conditions and especially in samples infected with *M. refringens*. However, increased hemocytic activity in individuals without the presence of the parasite may be induced by heat stress as a result of elevated temperatures [70]. Regarding the disruption, the thickness in the epithelial tissue and the inflammation in infected individuals, our results are in agreement with Carella et al. [71], who demonstrated the same results in infected individuals. The existence of all life stages of the parasite in both culture techniques (TC and LC) is confirming the implication of the parasite’s role in the mortalities and is in agreement with a previous study conducted in Thermaikos Gulf in 2006, verifying the existence of the parasite [58]. Despite the hemocytic infiltration in almost each sample, histopathological display confirmed the presence of the parasite as etiological agent of the degeneration process alongside the epithelial tissue.

Furthermore, the results of the present study concerning biochemical indicators are in line with other studies demonstrating the cumulative effects of multiple stressors [60,72,73]. Particularly, Hsp70 and Hsp90 demonstrated a similar pattern in each sampling in both culture techniques. Moreover, individuals infected by *M. refringens,* exhibited higher levels of expression of these genes than the non-infected ones. The higher expression pattern of both Hsps indicates the important roles they have in enabling cells to adapt to various stressors and maintaining normal cellular functions by counteracting misfolded cellular proteins [74]. Regarding the function of apoptosis, the ratio of pro-apoptotic (Bax) and pro-survival (Bcl-2) proteins, which is responsible for caspase activation, demonstrated the significant increase in *M. refringens* infected mussels in comparison with non-infected ones. Cleaved caspase levels followed the same pattern as the Bax/Bcl-2 ratio, exhibiting a “constant” pattern of apoptotic pathway stages in infected individuals. The aforementioned results lead to the conclusion that the activation of apoptosis is implicated in infection with *M. refringens* [75]. Similarly, TNF-a, which is produced to induce an inflammatory response, exhibited its involvement in *M. refringens*-infected individuals of *M. galloprovincialis* [76], although not in any seasonal pattern. The latter shows that heatwave as a sole stress factor does not trigger this stressor’s induction. Although Il-6, which is also an important mediator in immune responses, and its induction pattern confirm its implication in the inflammation process during *M. refringens* infection, the results of the present study are in contrast to Lattos et al. [60]. The latter did not demonstrate any significant differences in Il-6 levels in individuals facing multiple stressors in comparison with individuals infected only with one pathogen.

Apart from the post-translational level studies herein, mRNA expression was also determined in order to assess the transcription part of the protein expression process. Despite their role as environmental biomarkers, Hsps also play a vital role in several physiological processes such as gametogenesis, embryogenesis and metamorphosis in marine bivalve species [77,78]. In the case of mRNA expression, *hsp70* did not exhibit any significant differences between *M. refingens*-infected and non-infected animals. Specifically, non-infected animals exhibited higher expression levels. This fact can be attributed to their role in promoting immune responses involved in cellular protection against several stress factors including pathogens [79]. On the other hand, it should be highlighted that Hsps expression against stress factors varies and is in direct correlation with the involved stress factor [79]. Regarding gene expression related to the apoptotic pathway, no significant differences were observed regarding any of the different conditions examined: infection status, seasonality, culture technique. Our results depict a correlation of Hsp70-induced levels with reduced seasonal pattern expression of Bax [80]. The latter can be attributed to the fact that although apoptosis is induced by heat stress in marine invertebrates, Hsp70 has been shown to possess an anti-apoptotic function [80,81]. Similarly, irak4 and traf6 did not exhibit any seasonal pattern or any pattern with correlation with *M. refringens*.

The discrepancy between transcriptional and post-translational levels in the proteins measured in the present study can be attributed to the fact that mRNA levels do not accurately predict protein levels in eukaryotic cells. The latter is the case for our study, which exhibits a weak correlation between transcription and translation products’ levels, and therefore our results are in compliance with recent studies on eukaryotic cells that exhibit a discrepancy between mRNA and protein levels [82,83,84]. Most of the biochemical indicators measured herein are the result of versatile transcription and/or translation and post-translational regulation [85,86,87]. It could also be that the intense and sudden stress that mussels faced during temperature raise prohibited the defense mechanisms reflected in mRNA-measured gene expression from re-triggering leading to mortality events observed. Although the molecular mechanisms underlying widespread differences in translational efficiency are poorly understood, it is accepted that proteome regulation is the primary output of signaling pathways that connect cell physiology to internal and external environmental cues. Thus, gene-specific differences in translation are at least as important as transcriptional control in determining steady-state protein levels [88].

## 5. Conclusions

Since heatwaves are expected to be more often and severe due to ongoing climate change, drastic measures are required to ameliorate their impact on marine organisms. Moreover, the infection of marine organisms by pathogens that are favored by these heatwaves subsequently leads to a dramatic loss in marine production due to extensive mortalities. To this end, the present study aimed to identify the molecular and biochemical pathways, as well as histopathological findings induced under these extreme conditions, in order to provide insights for the future management of the marine and in general aquatic sectors. Although the heatwave increased all the above-mentioned parameters in *M. galloprovincialis* mussels, these responses as well as mortalities were enhanced in infected individuals under all culture techniques. The lightly and heavily infected by *M. refringens* individuals showed similarly increased biochemical responses in both LC and TC (compared with the non-infected mussels), revealing a stress response depending merely on the presence of the parasite. Therefore, our results evaluate for the first time the stress response of *M. galloprovincialis* under field conditions against this biotic factor, elucidating its biological impact. Because the concomitant effect of heatwave and *M. refingens* infection has led to devastating results regarding *M. galloprovincialis* in northern Greece’s farms in 2020 and 2021, it is of critical importance to fully address measures for the decrease or elimination of this phenomenon and restore marine production and the results described in this study are towards this scope. Finally, chronic exposure of mussels to heatwave phenomena is a field of research that should be investigated in future studies to incorporate such measures.

## Figures and Tables

**Table 1:** List of primers used in this study and their amplicon sizes and Genbank accession number.

Target Gene	Forward Primer (5′-3′)	Amplicon (bp)	GenBank Accession	Reference
	Reverse Primer (5′-3′)		No.	
*hsp70*	CGGAGGCAAGCCAAAACTAC	109	AB180909.1	Giannetto et al. [54]
	AGCCTCGGCAGTTTCTTTCA			
*bax*	CCAACAGGTCCACCATTAGAAC	151	KC545830.1	Estevez-Calvar et al. [55]
	CTCTTGGCCACAGTTAGGAATG			
*bcl-2*	AGATAACGGTGGTTGGCAAG TAACGCCATTGCGCCTAT	127	KC545829.1	Estevez-Calvar et al. [55]
*irak4*	TTTGAGGAAGATGCTAAACCTG	127	KC994891.1	Toubiana et al. [56]
	CAACTGAGAAACCCAAGAAAG			
*traf6*	GAAGGCTGTAAAGTGATAGAAGTT	135	KC994893.1	Toubiana et al. [56]
	CTGAGATAGATGATGAGGTAAGTC			
*β-actin*	CGACTCTGGAGATGGTGTCA	153	AF157491.1	Moreira et al. [57]
	GCGGTGGTTGTGAATGAGTA			
*EF-1α*	GATATGCGCCAGTCTTGGAT	223	AB162021	Moreira et al. [57]
	CTCATGTCTCGGACAGCAAA			

**Table 2:** Classification of *Marteilia refringens* infection.

Sampling Date	Infection Rate		
	Heavy	Moderate	Light
13 July 2022	+	−	−
25 July 2022	+	−	+
1 August 2022	−	−	+

(+) represents presence and (−) absence of *Marteilia refringens.*

## Data Availability

Available on reasonable request from the corresponding authors.

## Supplementary Materials

The following supporting information can be downloaded at: https://www.mdpi.com/article/10.3390/ani12202805/s1, Figure S1: The complete original immunoblots shown in Figure 7 are presented in order below. The individual parts comprising Figure 7 are specified using black boxes; Figures S2 and S3: The complete original immunoblots shown in Figure 9 are presented in order below. The individual parts comprising Figure 9 are specified using black boxes; Figure S4: The complete original immunoblots shown in Figure 11 are presented in order below. The individual parts comprising Figure 11 are specified using black boxes.
