# Supplementary material for: Are Marine Heatwaves Responsible for Mortalities of Farmed Mytilus galloprovincialis? A Pathophysiological Analysis of Marteilia Infected Mussels from Thermaikos Gulf, Greece
Source: Animals (Basel). 2022 Oct 17;12(20):2805. doi: 10.3390/ani12202805 (PMC9597814; doi:10.3390/ani12202805)

Are marine heatwaves responsible for mortalities of farmed *Mytilus galloprovincialis* mussels? A case study in Thermaikos gulf, Greece

Athanasios Lattos[1*], Dimitrios K. Papadopoulos[1], Kostantinos Feidantsis[1], Ioannis A. Giantsis[2], Dimitrios Karagiannis[3] and Basile Michaelidis[1*]

Figure S1: The complete original immunoblots shown in Figure 7 are presented in order below. The individual parts comprising Figure 7 are specified using black boxes.

Cropped blots in main paper

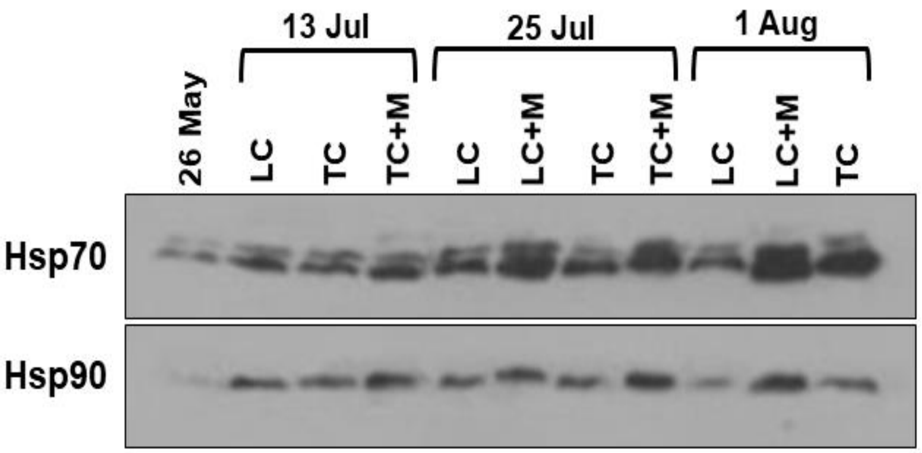

Original blots

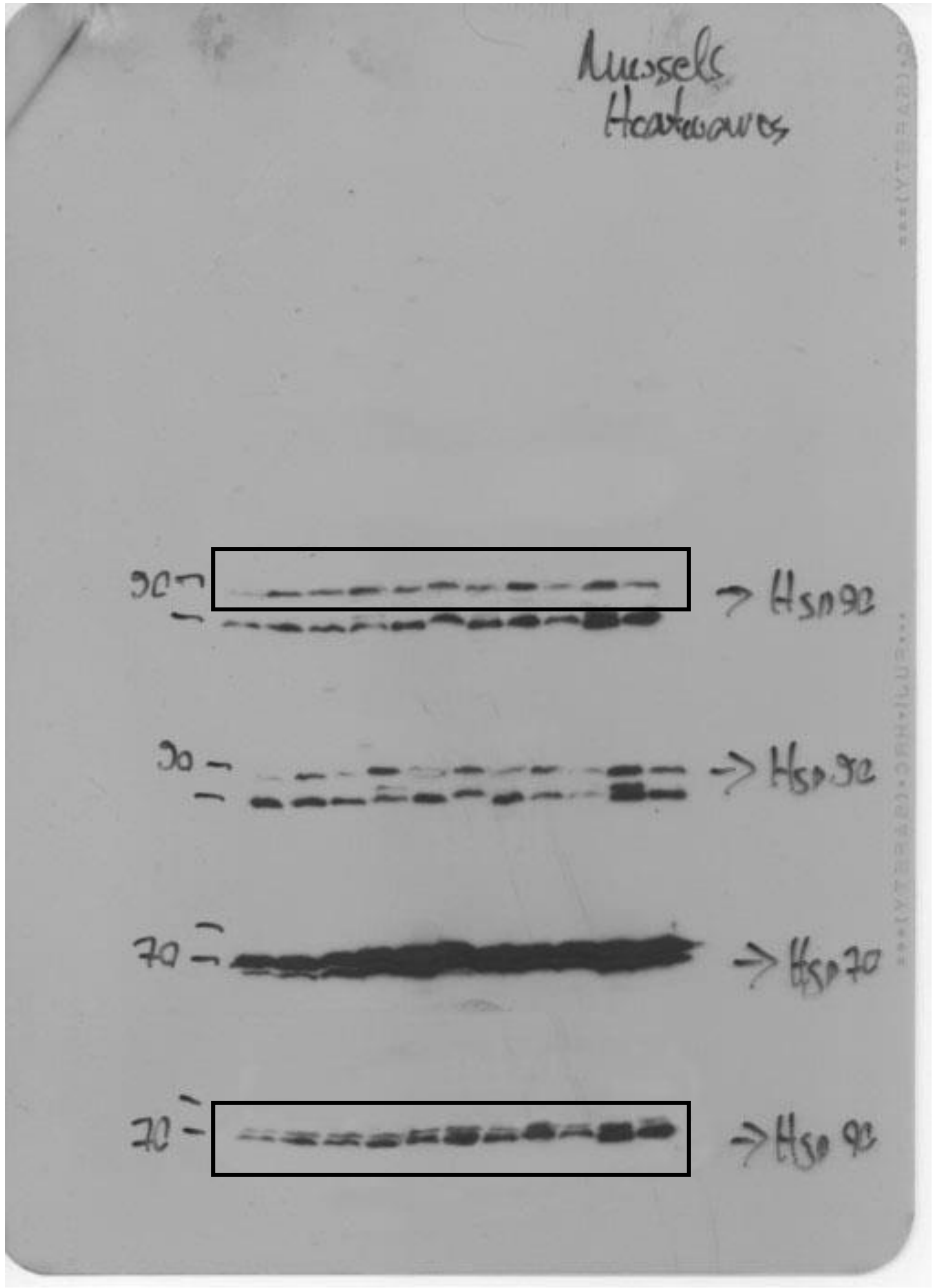

Figure S2: The complete original immunoblots shown in Figure 9 are presented in order below. The individual parts comprising Figure 9 are specified using black boxes.

Cropped blots in main paper

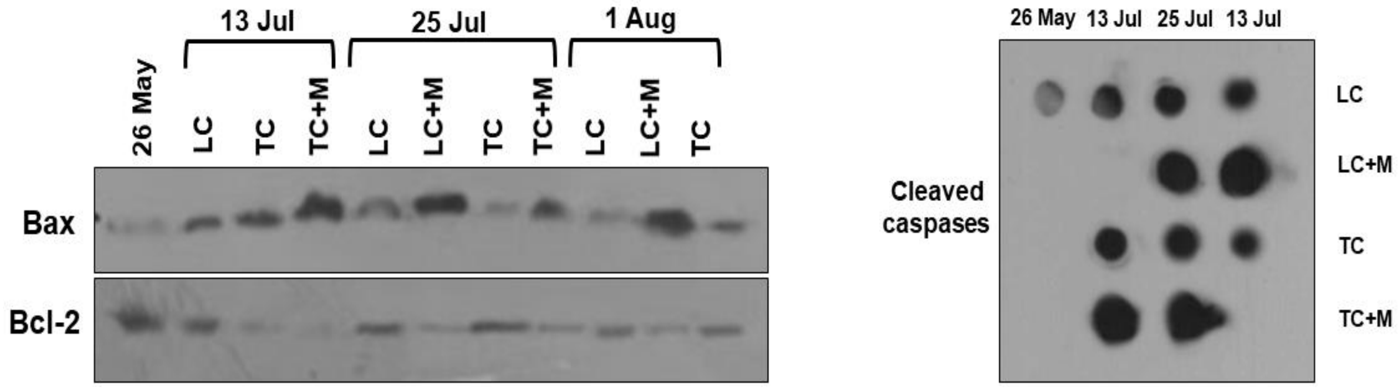

Original blots

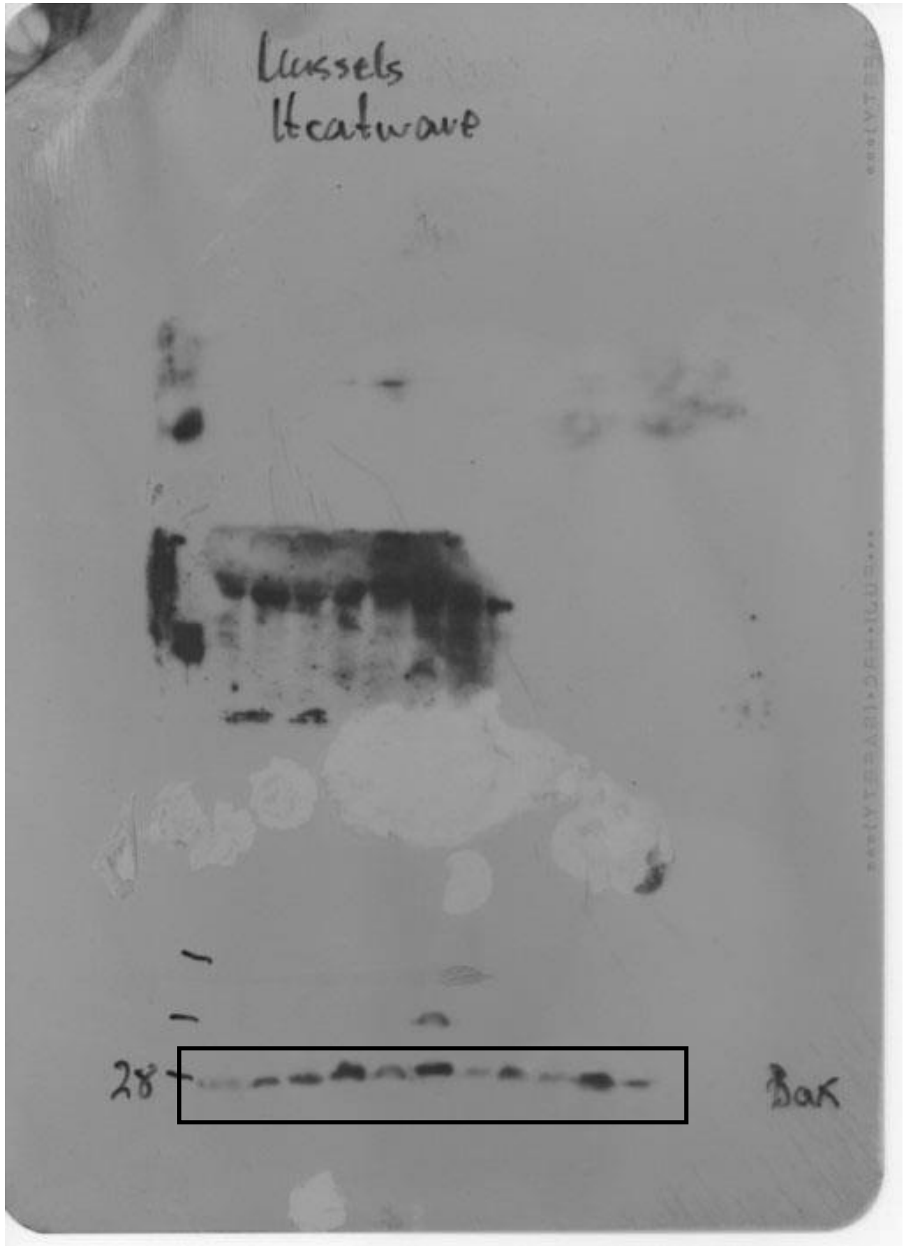

Figure S3: The complete original immunoblots shown in Figure 9 are presented in order below. The individual parts comprising Figure 9 are specified using black boxes.

Cropped blots in main paper

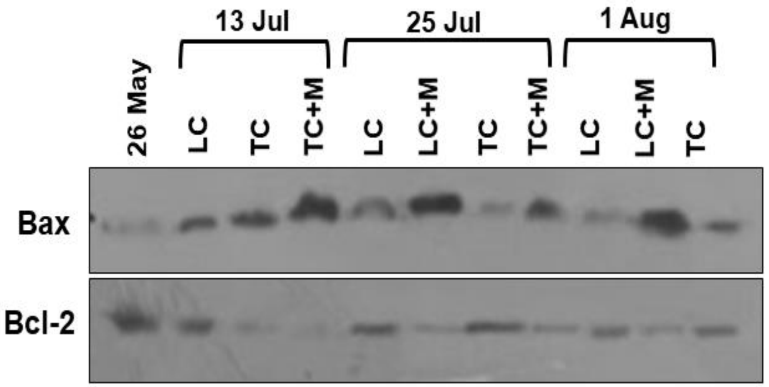

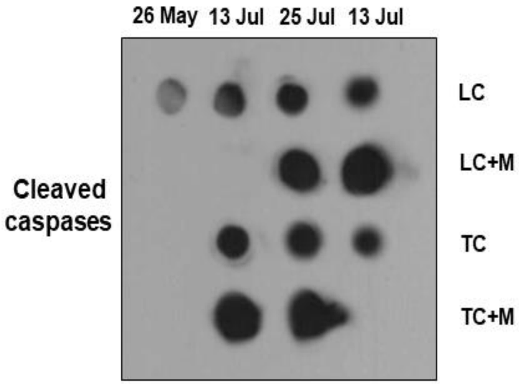

Original blots

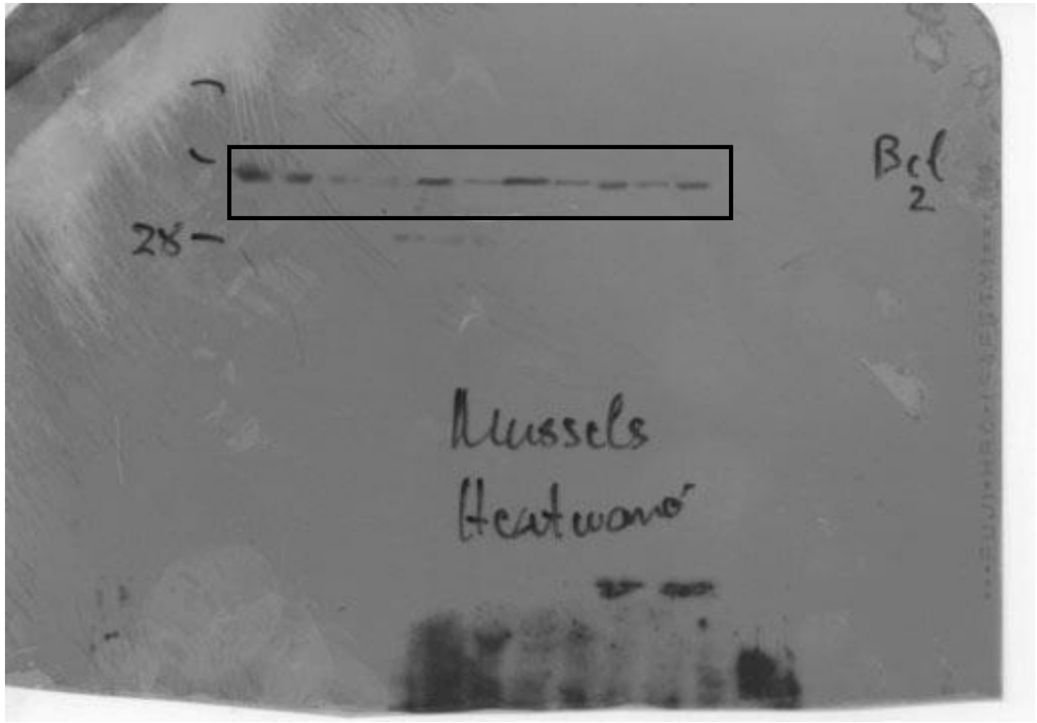

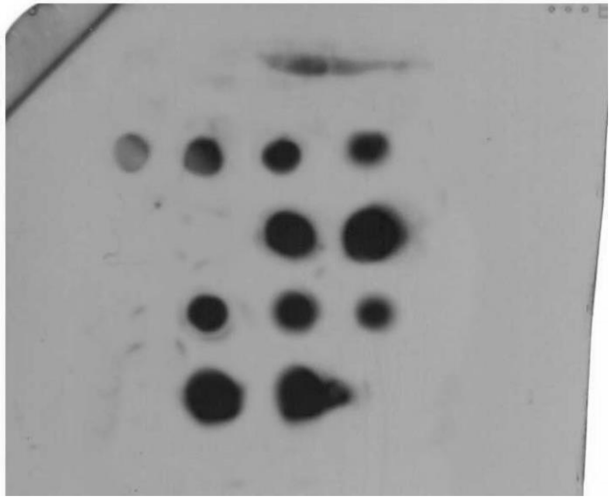

Figure S4: The complete original immunoblots shown in Figure 11are presented in order below. The individual parts comprising Figure 11 are specified using black boxes.

Original blots

Cropped blots in main paper

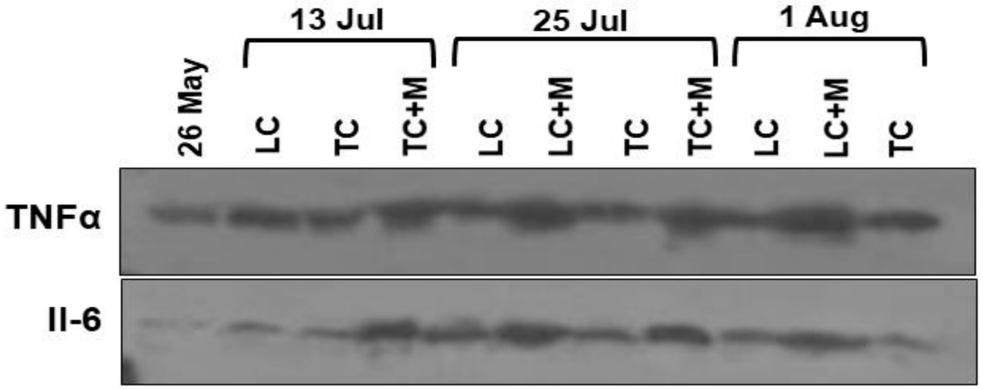

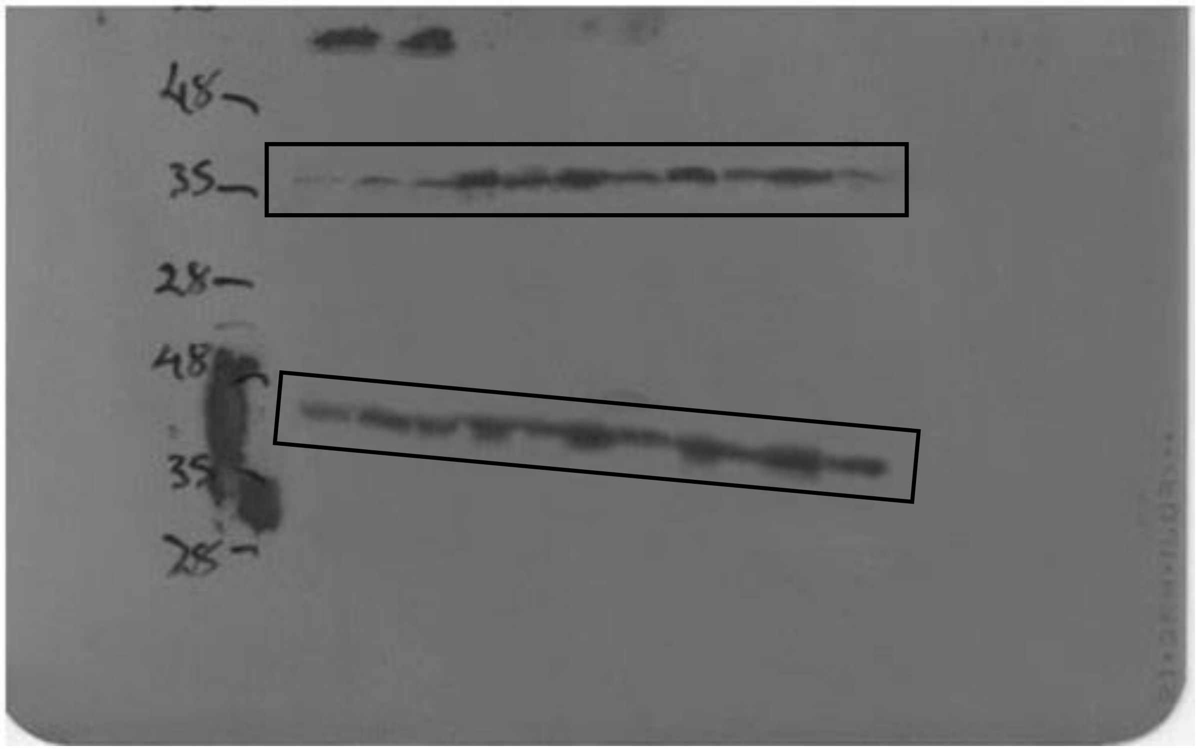

Supplement: Supplementary file 1 [file animals-12-02805-s001.zip › animals-1917486-supplementary.pdf]
